# Forelimb stripe coloration signals age, but not physiological health, in painted turtles

**DOI:** 10.1007/s00265-025-03679-0

**Published:** 2025-12-29

**Authors:** Jessica M. Judson, Luke A. Hoekstra, Kaitlyn G. Holden, Anne M. Bronikowski, Fredric J. Janzen

**Affiliations:** 1 Department of Ecology, Evolution, and Organismal Biology, Iowa State University, Ames, IA 50011 USA; 2 Department of Biology, University of Wisconsin – La Crosse, La Crosse, WI 54601 USA; 3 Department of Integrative Biology, Oklahoma State University, Stillwater, OK 74078 USA; 4 Departments of Fisheries and Wildlife & Integrative Biology, W. K. Kellogg Biological Station, Michigan State University, Hickory Corners, MI 49060 USA

**Keywords:** Color, Physiology, Stress, Immune function, Reptile, Aging

## Abstract

Variation in aspects of carotenoid-based coloration reflects physiological health in many taxa. Painted turtles (*Chrysemys picta*) have brightly colored limb stripes produced by carotenoids, but the function of this color and its relation to physiological function are unknown. We tested the hypothesis that variation in painted turtle forelimb stripe coloration is associated with variation in physiological health – including glucocorticoids and both innate and adaptive immune function. We assessed adult painted turtles of both sexes across a range of sizes/ages from a long-studied population. We found that body size – a strong indicator of age in this species – was associated with changes in forelimb stripe color, such that larger turtles have brighter, more colorful stripes with increased contrast from the background forelimb color. We further found that a number of physiological health measures either changed with size, or differed between sexes, yet physiology measures were not associated with stripe color. Thus, forelimb stripe coloration is not a direct honest signal of quality. Secondarily, we provide a suite of protocols for future use to understand physiological variation of reptiles in the wild. We discuss our results in the context of signaling, physiological health, aging, and sex-specific life histories.

**Supplementary Information:**

The online version contains supplementary material available at 10.1007/s00265-025-03679-0.

## Significance statement

Carotenoid-based coloration is an honest signal of quality in many vertebrates, and many studies have detected relationships between physiological state and coloration of ornaments used to communicate with conspecifics. However, the relationships between coloration and physiological fitness in many non-avian vertebrates remain untested, and studies that assess a suite of physiological measures including stress levels and immune function are rare. We assessed relationships among measures of physiology, size (age), sex, and forelimb stripe color in the colorful painted turtle. We found that stress response measures and immune function were not associated with forelimb coloration. Instead, brightness and hue increased with increasing body size. This study provides the foundation for future work to assess whether painted turtle color is a signal of health to conspecifics, and whether such coloration plays a role in painted turtle fitness.

**Supplementary Information:**

The online version contains supplementary material available at 10.1007/s00265-025-03679-0.

## Introduction

Color is important for conspecific and heterospecific communication, thermoregulation, and camouflage across vertebrates (Cuthill et al. 2017). Some of the most dramatic colors in nature are attributed to sexual selection driving the evolution and maintenance of brightly colored ornaments used to attract mates (e.g., Hill and McGraw 2006). One mechanism that would promote the evolution of colorful ornaments or patterns used for attracting mates is if coloration is an honest signal of overall mate quality (Andersson 1986). If color production is costly or is a reflection of physiological links between condition and color (Weaver et al. 2017; Hill et al. 2023), an individual’s coloration should reflect its physiological state.

Carotenoid-based coloration is perhaps the most well-studied example of a signal that can honestly convey individual mate quality (Svensson and Wong 2011; Hill et al. 2023). Carotenoids cannot be synthesized by animals and must be obtained from the diet, yet they are linked to a variety of physiological processes in addition to color, including mitochondrial function (Hill et al. 2019) and immune function (Zhang et al. 2023). If carotenoids are costly to allocate to signaling instead of physiological maintenance (the resource trade-off hypothesis; Lozano 1994), or if carotenoid coloration is inherently tied to fundamental physiological processes (the shared pathway hypothesis; Hill et al. 2023), a relationship between carotenoid ornament color and physiological health is expected. Indeed, relationships between baseline stress biomarkers and coloration (Fitze et al. 2009) or immune function and coloration (Desprat et al. 2017) have been found across many organisms (reviewed in Svensson and Wong 2011; but see Weaver et al. 2018b). Because physiological processes such as responses of the hypothalamo-pituitary-adrenocortical axis (hypothalamic-pituitary-interrenal, or HPI, axis in reptiles) and immune function are themselves linked (Padgett and Glaser 2003), coloration may reflect mate quality in multiple axes of physiological health (Hill 2014). Despite continued interest in the ecological role of animal coloration (Caro et al. 2017) and the calls to measure multiple aspects of individual physiological function (Svensson and Wong 2011), relatively few studies have analyzed multiple aspects of physiology and their comprehensive relationships with color.

In this study, we tested an array of measures associated with function of the HPI and immune axes of physiological state and coloration of a reptile in an integrative and comprehensive approach. The painted turtle (*Chrysemys picta*) has a widespread distribution in slow-moving freshwater systems across North America. This species is named for its colorful plastrons and red, orange, and yellow stripes on the limbs and head, which are produced by carotenoids (Steffen et al. 2015). The role of stripe coloration is unknown, but it may be used as an honest signal to conspecifics and potential mates. Male painted turtles perform an underwater courtship display to females before mating, called titillation, during which a male swims facing a female and strokes the sides of a female’s head with his foreclaws (Ernst and Lovich 2009). Carotenoid availability in the diet impacts color of painted turtle stripes, which may indicate a trade-off in carotenoid allocation to attract mates dependent on environmental availability (Steffen et al. 2019, 2021). Further, in a closely related turtle species, the red-eared slider (*Trachemys scripta*), chin stripes darkened with a direct immune challenge (Ibáñez et al. 2014). Using adult painted turtles, we tested the hypothesis that levels of baseline stress indicators and immune function influence forelimb stripe coloration. We measured baseline circulating stress indicators (corticosterone, glucose, and heterophil: lymphocyte ratios), innate immune function (circulating natural antibodies, complement-mediated cell lysis ability, and bactericidal competence of plasma), and adaptive immune function (B- and T-lymphocyte proliferative ability to three mitogens) to understand the effects of multiple axes of physiology on coloration in a reptile. We predicted that physiology measures would be strongly correlated, that turtles with higher quality (lower stress levels and increased immune function) would have greater forelimb stripe luminance and colorfulness, and that the stripes of these individuals would have a greater contrast to that of the darker forelimb background color from the perspective of a turtle. We also predicted that sex and body size, which is a proxy for age (Hoekstra et al. 2018), are associated with forelimb stripe color, given the proposed shift in mating strategy from courtship to coercion with increasing body size in male painted turtles (Moldowan et al. 2020b).

## Methods

### Husbandry and physiology sampling

We captured painted turtles for this study using hoop nets in July 2014 from the Thomson Causeway Recreation Area (TCRA) in Thomson, IL in the United States. We transported these turtles ~ 300 km directly west to Iowa State University (ISU), where we housed turtles for brumation (Supplementary Material). We followed all applicable ISU IACUC guidelines for the care and use of animals in this study. In April 2016, we seeded three semi-natural experimental ponds at the ISU Horticulture Research Station with 58 painted turtles (36 males, 22 females) which varied in population density (29, 17, or 12 turtles, respectively). Turtles lived in the ponds during the summer months, consuming aquatic plants, anurans, and invertebrates that colonized the ponds along with supplementary Mazuri^®^ Aquatic Turtle Diet.

In July 2016, at the conclusion of the nesting season, we drained all experimental ponds and removed the turtles. This period of time falls in the window of breeding activity, when females are not gravid and males are attempting mating, in at least some populations (Gist et al. 1990; Moldowan et al. 2020a). We measured plastron length and obtained a blood sample from the caudal vein of each turtle with a heparin-rinsed syringe within 10 min of capture to assess baseline measures of circulating corticosterone (CORT) and glucose (Polich 2016). We aliquoted whole blood (50µL) into tubes with 50µL of AIM V serum-free lymphocyte cell medium for lymphocyte proliferation assays (Palacios et al. 2013) and made a blood smear stained with Wright Giemsa for differential cell counts. We centrifuged the remaining whole blood (from 30 to 150µL) and separated the plasma into two aliquots for CORT and immune assays before flash freezing in liquid nitrogen and storing at −80 °C. Given we needed to connect blood samples to individual turtles and our study involved focal animals in an experimental setting, it was not possible to record data blind. However, all physiology assays described below were randomized, such that individual samples were run in no particular order with respect to sex or size of the turtle.

### Color analysis

One week following removal of turtles, we took RAW-formatted photographs (tripod-mounted Canon EOS Digital Rebel XSi camera and EF-S18-55 mm lens) of each turtle’s cranial region under controlled incandescent lighting and included a grey standard (18% reflectance; Insignia NS-DWB3M) in every photograph. We took two photographs of each turtle at slightly different angles and checked photographs for overexposure before performing Quantitative Color Pattern Analysis (QCPA) in micaToolbox v. 2.3 (Troscianko and Stevens 2015; van den Berg et al. 2020; Supplementary Material) within ImageJ v. 1.54 g (Schneider et al. 2012). micaToolbox linearizes photographs using a grey standard and calculates reflectance values, which are then converted to cone-catch values using a model that accounts for the spectral sensitivities of the species of interest. These cone-catch values can then be used to analyze differences in luminance (the lightness and brightness), chromaticity (“colorfulness”), and achromatic and chromatic contrast elicited by and between specific regions of interest (ROIs) on the organism.

To analyze painted turtle color as perceived by a turtle, we created a cone-catch model of painted turtle vision using the chart-based method of micaToolbox. We photographed a X-Rite ColorChecker Passport Photo 2 (X-Rite; Michigan, USA) with the aforementioned camera and lens under two conditions: outdoor sunlight, and incandescent lighting, under which the turtle photographs were taken. We then developed cone-catch models under both lighting conditions using a custom spectral sensitivity profile created from two turtle species. We obtained a long wavelength (LW) spectral sensitivity curve from a flicker photometry study in adult painted turtles (Graf 1967) and medium wavelength (MW), short wavelength (SW), and double cone spectral sensitivity curves from intracellular retinal preparations of adult red-eared slider turtles (Ohtsuka 1985), as there were no spectral sensitivity curves in those wavelength ranges for painted turtles. Though painted turtles also likely have cones sensitive to ultraviolet (UV) wavelengths given their presence in the closely related red-eared slider turtle (Zana et al. 2001), we did not have the capability to photograph UV reflectance measures in this study, and exclude any spectral sensitivities in the UV range. We found close agreement in the cone-catch values across wavelength channels between the two different light sources (Supplementary Material); thus, while we use the cone-catch model generated using a chart photograph taken under incandescent lighting conditions for QCPA, the results of this work should be applicable to natural lighting conditions as well.

**Fig. 1:**
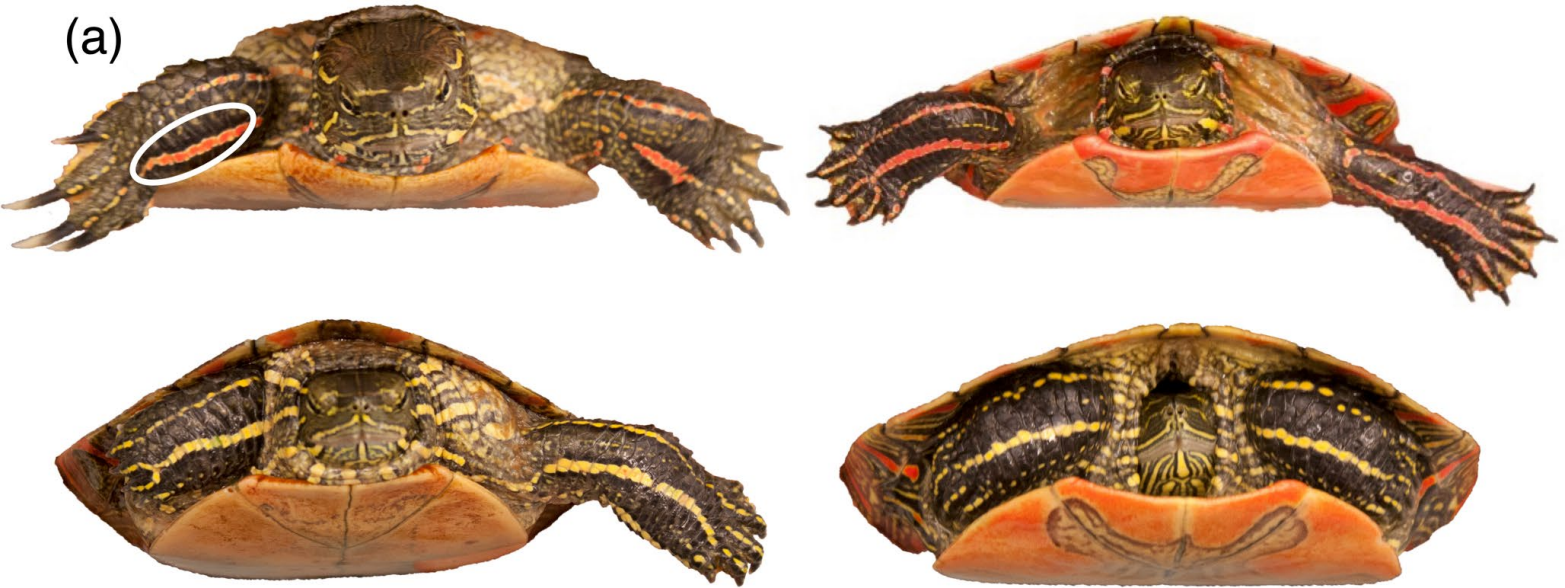
Four example images of turtles from this study showing the variation in forelimb stripes from the perspective of a human viewer. **a**) shows the region of interest (ROI) measured in this study, the forelimb stripe from elbow to claw. Backgrounds for all images were removed using Adobe Photoshop CC 2018

We measured color characteristics of a specific ROI on each turtle, the part of the turtle’s forelimb from the claw to the elbow which contains a red, orange, or yellow stripe (Fig. 1a, S1). These stripes are visible to conspecifics during male courtship displays and aggression (Ernst and Lovich 2009; Moldowan et al. 2020b), and thus may serve as a signal of fitness to potential mates or competitors. All settings used in QCPA can be found in the Supplementary Material. Clustering of the forelimb ROI resulted in two clusters of color, the stripe and the background color of the forelimb surrounding the stripe. Analysis of these clusters produced average cone-catch values for each class of wavelength (LW, MW, SW, double cones) and *D*_max_. Stripe luminance was calculated as the average cone-catch values of the double cones, as the function of double cones is hypothesized to be more important for luminance perception than for color perception in birds (e.g., Vorobyev et al. 1998) and lizards (Olsson et al. 2013), and non-avian and avian reptiles share many similarities in visual parameters related to color vision (reviewed in Osorio 2019). To describe chromaticity of the stripe, we used *D*_max_, which is a measure of the maximum possible chromaticity (relative cone-catch value difference between all possible cone type comparisons) that could be elicited in a hypothetical opponent process (Endler and Mielke 2005; van den Berg et al. 2020). Using *D*_max_ to describe color may be appropriate when the opponency system is unknown or violates assumptions of the Receptor Noise Limited Model (RNL; Vorobyev and Osorio 1998; van den Berg et al. 2020), as is possibly the case with pond turtle vision (Rocha et al. 2008). Larger values of *D*_max_ indicate greater maximum possible chromaticity (colorfulness) of a stripe.

While turtles may violate assumptions of the RNL model, they may also violate assumptions of *D*_max_. More than two cones may be involved in a given opponent channel, and the high number of opponent channels found in another species of turtle suggests this may be a possibility (Rocha et al. 2008). Additionally, there are currently no methods to easily compare the chromaticity of two regions with *D*_max_, which means it can be difficult to interpret the ecological relevance of the signal to a receiver. Therefore, we also measured the achromatic (∆L) and chromatic (∆S) contrast between the stripe region and background region of the forelimb ROI using RNL methods in micaToolbox (Supplementary Material). These values can be interpreted similarly in that higher values indicate a greater contrast in brightness (∆L) or hue (∆S), and thus greater ability of a turtle to distinguish the stripe from the background.

We assessed repeatability (intraclass correlation coefficient, Lessells and Boag 1987) of average cone-catch values, *D*_max_, ∆L, and ∆S using the two photos of each turtle in R v. 4.4.2 (R Core Team 2024). Measures were repeatable (79%−91%) across photographs; thus, we averaged each of these measures across the two photographs of each turtle to produce final color variables used in statistical analyses. We assessed correlations and performed a principal components analysis (PCA) of color variables to assess axes of color variation across turtles. Variables were centered and scaled before performing PCA with ‘prcomp’ in R.

### Physiological measures

#### Baseline stress indicators: corticosterone, glucose, and heterophil: lymphocyte ratios

The release of CORT induces increases in circulating glucose (Landys et al. 2006), increases in heterophils (neutrophils in mammals), and decreases in lymphocytes in the bloodstream to prepare the organism to appropriately respond to stressors (reviewed in Goessling et al. 2015). We used a double-antibody radioimmunoassay (*N* = 58) to quantify concentration of circulating plasma CORT following previously described protocols validated in painted turtles (Supplementary Material; Refsnider et al. 2015; Polich 2016). We measured the baseline concentration of circulating glucose (Mg/dL) using 1.5µL blood plasma with a FreeStyle Lite^®^ glucometer (Abbott Diabetes Care, Alameda, CA) and FreeStyle Lite^®^ test strips (*N* = 56; Gangloff et al. 2017). We analyzed heterophil: lymphocyte (H: L) ratios via stained blood smears by identifying 100 leukocytes at 1000x magnification and counting the number of heterophils and lymphocytes within those leukocytes (*N* = 55; Gangloff et al. 2017). Under chronic stress conditions, a large H: L ratio is produced by glucocorticoids mobilizing lymphocytes into tissues and out of the bloodstream, while heterophils are increased in the bloodstream (Davis et al. 2008).

#### Innate immune function: bactericidal competence of plasma, natural antibodies, and lysis

The bactericidal competence (BC) of plasma measures constitutive innate immune function. Turtles with increased innate immune function are characterized by a high bacterial killing capacity, or competence, while individuals with depressed immune function may exhibit lower bactericidal competence (Matson et al. 2006). We assessed BC of plasma (*N* = 55) according to Refsnider et al. (2015) with a few modifications (Supplementary Material). We detected a batch effect due to decreased survivorship of the *E. coli* working stock over time, and thus standardized BC within each batch (3 batches over 3 days).

Natural antibodies (NAbs) and complement-mediated lysis (CL) are two additional measures of constitutive innate immunity; high levels of NAbs and CL activity indicate a higher level of innate immune defense (Matson et al. 2005). We assessed these immune measures using a haemolysis-haemagglutination assay (*N* = 53) modified from Matson et al. (2005) for use in painted turtles (Supplementary Material; Schwanz et al. 2011; Refsnider et al. 2015).

#### Adaptive immune function: lymphocyte proliferative ability

Lymphocyte proliferation assays measure an organism’s adaptive immune function by assessing the activation and proliferation of B- and T-lymphocytes in response to a mitogen; increased proliferation indicates a stronger immune response (Palacios et al. 2013). We gauged lymphocyte proliferation ability with a whole-blood mitogenic stimulation assay (Palacios et al. 2013) performed within 24 h of blood collection (*N* = 55). We used two T-cell mitogens, concanavalin A (ConA) and phytohemagglutinin (PHA), and one B-cell mitogen, lipopolysaccharide (LPS). Detailed methods can be found in Palacios et al. (2013; Supplementary Material). The proliferative ability of lymphocytes is expressed as a stimulation index (SI; Palacios et al. 2013), which is a ratio that compares mean counts per minute of mitogen-stimulated samples and non-stimulated controls. To control for differences in the initial number of lymphocytes in each sample, we estimated total leukocyte counts using the indirect Phloxin B method (Campbell and Ellis 2007) with 0.1% phloxin stain (Vetlab Supply, Palmetto Bay, FL) and hemocytometer (Palacios et al. 2013), and then we used H: L slide preparations to estimate the number of lymphocytes in total leukocyte counts for each turtle. To correct the SI for starting number of lymphocytes, we performed a linear regression of starting number of lymphocytes versus SI for each mitogen. We used the residuals from those models as values for SI of the three mitogens.

### Statistical analyses

We used R v. 4.4.2 (R Core Team 2024) for all statistical analyses described here. There were no strong outliers in the physiology variables, with the exception of one individual for SI_ConA_ and two individuals for SI_PHA_ and SI_LPS_. The two outliers for SI_PHA_ and SI_LPS_ were turtles that exhibited high proliferation responses to both mitogens, and thus may be the result of biological differences. All models were run both removing or including the outliers, and in the one case where model results changed with their exclusion, we discuss both model outcomes. We first calculated correlations among all physiological variables to assess our prediction that baseline stress measures and immune function measures are associated with one another. We used Kendall’s rank correlation coefficient (Kendall 1938) given the presence of skewed distributions and putative outliers in physiology measures, used only complete observations, and calculated *P* values with Holm’s correction (Holm 1979) using the ‘corr.test’ function of the “psych” R package (Revelle 2024). We then performed a PCA to assess major axes of variance in physiology measures among turtles. We centered and scaled all variables except BC, which was already centered and scaled to account for batch effects.

To understand the relationships among color, body size, and sex in painted turtles, we first standardized plastron length by sex (zPL) using a sex-specific z-transformation, as female painted turtles attain larger body sizes than males (Hoekstra et al. 2018). We then performed general linear models of forelimb stripe luminance, chromaticity (*D*_max_), achromatic contrast (∆L), or chromatic contrast (∆S) with zPL, sex, and the interaction of zPL and sex as predictors using ‘lm’ in R. We included the interaction between sex and zPL because recent research suggests that mating strategy of male painted turtles may shift from courtship to coercion with increasing body size (Moldowan et al. 2020b). We assessed the relationship between physiology measures and color measures using general linear models with ‘lm’. We calculated F statistics and assessed statistical significance from model outputs using type III sums of squares analysis of variance in the “car” package (Fox and Weisberg 2019). We assessed model assumptions with “DHARMa” (Hartig 2022) and “car”.

To test the influence of separate immune and stress measures, we ran two sets of models including either the baseline stress measures or the immune measures with stripe luminance, stripe chromaticity, achromatic contrast, or chromatic contrast as the dependent variable. For all models, we included the interaction between zPL and sex and the fixed effects of sex, the pond in which each turtle was kept, and zPL. Thus, the models for stress measures included pond, sex, zPL, the interaction of sex and zPL, CORT, glucose, and H: L ratio. The models for immune measures included pond, sex, zPL, the interaction of sex and zPL, standardized BC, NAbs, CL, SI_ConA_, SI_PHA_, and SI_LPS_. When statistically significant (i.e., *P* < 0.05), marginal means and pairwise contrasts among factors were estimated from models using “emmeans” (Lenth 2023). Finally, we assessed the relationship between physiology measures, sex, and zPL, which is a proxy for age in painted turtles, using general linear models (Hoekstra et al. 2018). We graphed all plots using “ggplot2” (Wickham 2016). All data used for building turtle visual models, raw photographs, multispectral images, raw physiology measures, R scripts, and final color variables can be found in the repository (see Data Availability).

## Results

We found considerable variation in color of painted turtle forelimb stripes from the perspective of a human viewer (Fig. 1), and analyses of color from a turtle’s visual perspective also found variation in luminance and chromaticity of forelimb stripes. Further, all values for both achromatic and chromatic contrast were greater than 3, which suggests that in all cases the stripe could be distinguished from the background forelimb color in a natural setting by a turtle (Vorobyev and Osorio 1998). Cone-catch values of all wavelengths measured (LW, MW, SW, double cones) were all significantly positively correlated (Table S1). The first two PCs of the PCA of cone-catch values accounted for 86% and 12% of the variance in color measures, respectively, with the first PC demonstrating the strong positive correlations among cone-catch values (Fig. S2). We also found that stripe-specific measures of color (luminance and chromaticity) were significantly correlated with measures of achromatic and chromatic contrast between stripe and background forelimb color (Pearson’s *r* = 0.54 and 0.87, respectively; Table S1). This correlation indicates that, although turtle vision may be violating model assumptions for both *D*_max_ and chromatic contrast calculated with the RNL model, the results of both methods are similar. As results of stripe-specific measures and measures of contrasts were the same in almost all models tested, we include results of the contrasts here and include results of stripe-specific measures of luminance and chromaticity in the Supplemental Material.

Though we expected physiology measures to covary within individuals, there were no significant correlations among any physiology measures (Table S2). The largest correlation coefficient (0.44) was between NAbs and BC, followed by lymphocyte proliferative responses to PHA and LPS. In agreement with low correlation coefficients, the PCA did not detect axes that accounted for a large proportion of variance (Fig. S3). Almost all variables loaded positively on PC1 (26% of variance), but some variables (CORT, NAbs, BC, CL) loaded more strongly than others (glucose, H:L; Fig. S4).

**Fig. 2:**
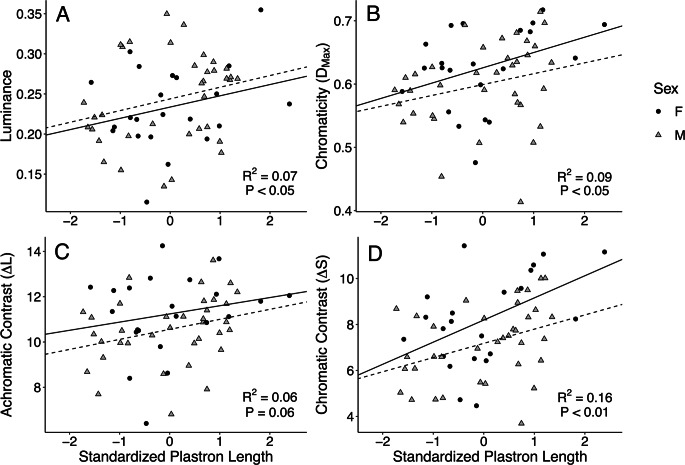
Positive relationships of size and (**A**) luminance, (**B**) chromaticity (*D*_max_), (**C**) achromatic contrast (∆L), or (**D**) chromatic contrast (∆S) of painted turtle forelimb stripes. Plastron length was standardized by sex. *R*^2^ and *P* values are from simple linear regressions of each color measure versus standardized plastron length of all measured turtles. Solid lines reflect the simple linear regression including only females, and dashed lines reflect males

We hypothesized that painted turtle forelimb stripe color would vary with size (measured as standardized plastron length) and sex. The interaction between sex and size was not significant for any color measure (Table S3), and when this interaction term was excluded from the models, we found that size was positively associated with luminance (*P* = 0.047) chromaticity (*P* = 0.023), achromatic contrast (*P* = 0.055), and chromatic contrast (*P* = 0.001; Fig. 2, Table S4). This pattern of increasing brightness and colorfulness (hue) with increasing size was consistent for both males and females. Females also displayed greater chromatic contrast than males (*P* = 0.025; Fig. 2; Table S4).

We also hypothesized that baseline stress levels and immune function predict forelimb stripe coloration. However, we found no significant relationships among any of the physiology measures and either of the stripe-specific measures, luminance or chromaticity (Table S5, S6), or achromatic contrast (Tables 1 and 2). The only significant relationship of any physiology measure and color was the negative relationship between lymphocyte stimulation index in response to lipopolysaccharide (LPS) and chromatic contrast (*P* = 0.022; Table 2; Fig. S5). This relationship is entirely driven by an outlier, and removal of this outlier causes the relationship to no longer be significant or negative in direction. Only size (zPL) and sex showed consistent relationships to color measures in the models. The relationships between size and color measures were positive, as in the models including only size and sex above. In the models including stress measures, females had slightly greater stripe chromaticity and chromatic contrast than males, and in the model including immune measures, females had less stripe luminance than males.

**Table 1:** Relationship between baseline stress measures and painted turtle forelimb achromatic or chromatic contrast

Dependent Variable	Source of Variation	df	F	*P*
Achromatic Contrast (∆L)	Pond	2, 44	1.5022	0.2338
	Sex	1, 44	0.4914	0.4870
	zPL^a^	1, 44	2.5431	0.1179
	CORT^b^	1, 44	0.2864	0.5952
	Glucose	1, 44	1.3726	0.2477
	H: L^c^	1, 44	0.0063	0.9370
	Sex * zPL	1, 44	0.0045	0.9468
Chromatic Contrast (∆S)	Pond	2, 44	2.2770	0.1145
	Sex	1, 44	3.3724	0.0731
	zPL	1, 44	10.6478	**0.0021**
	CORT	1, 44	0.0311	0.8608
	Glucose	1, 44	0.1426	0.7075
	H: L	1, 44	1.8599	0.1796
	Sex * zPL	1, 44	0.1088	0.7431

df is degrees of freedom. P < 0.05 in bold. ^a^ plastron length standardized for each sex; ^b^ corticosterone concentration; ^c^ heterophil: lymphocyte ratio

**Table 2:** Relationship between immune function measures and painted turtle forelimb achromatic or chromatic contrast

Dependent Variable	Source of Variation	df	F	*P*
Achromatic Contrast (∆L)	Pond	2, 33	2.3325	0.1128
	Sex	1, 33	0.2931	0.5919
	zPL^a^	1, 33	0.3480	0.5593
	NAbs^b^	1, 33	0.2865	0.5960
	CL^c^	1, 33	0.0180	0.8941
	BC^d^	1, 33	0.0069	0.9341
	ConA^e^	1, 33	1.8033	0.1885
	PHA^f^	1, 33	0.2531	0.6183
	LPS^g^	1, 33	1.7840	0.1908
	Sex * zPL	1, 33	0.1082	0.7443
Chromatic Contrast (∆S)	Pond	2, 33	0.4403	0.6476
	Sex	1, 33	0.2548	0.6170
	zPL	1, 33	6.2021	**0.0180**
	NAbs	1, 33	0.4332	0.5150
	CL	1, 33	0.0293	0.8652
	BC	1, 33	0.0530	0.8193
	ConA	1, 33	4.0942	0.0512
	PHA	1, 33	2.5232	0.1217
	LPS	1, 33	5.7712	**0.0221**
	Sex * zPL	1, 33	1.4509	0.2370

df is degrees of freedom. P < 0.05 in bold. ^a^ plastron length standardized by sex; ^b^ natural antibodies; ^c^ complement-mediated lysis; ^d^ bactericidal competence standardized by batch; ^e^ residuals of models of lymphocyte proliferative responses to concanavalin A, ^f^ phytohemagglutinin, and ^g^ lipopolysaccharide against starting number of lymphocytes

Finally, we assessed whether measures of physiological health changed with size or sex. We found that two measures of physiological health (CORT and natural antibodies) decreased with increasing size (a proxy for age) for both sexes (Table S7). We also found sex-specific differences in complement-mediated lysis and H: L ratios, which were both higher in males than females. While physiology did not predict coloration, size and sex predicted some measures of baseline stress and immunity as well as measures of color. This presents an intriguing question as to whether physiological health may influence coloration through indirect (age-related) pathways. As this is not the focus of this study, we do not consider this hypothesis here further.

## Discussion

While we hypothesized that physiology would predict coloration of painted turtle forelimb stripes, a potentially honest signal of quality to conspecifics, we found no strong relationships between any measured aspects of baseline stress indicators or immune function and color of forelimb stripes. Instead, physiology measures were largely uncorrelated, and the strongest predictors of forelimb stripe coloration were body size and sex. Thus, variation in coloration of forelimb stripes may not directly reflect differences in physiological health. However, our results are restricted to the visible spectrum; thus, any relationships between variation in coloration in the UV spectrum and physiology remain unknown. These relationships may be important given the UV sexual dichromatism reported in painted turtles (Steffen et al. 2021), and all cone types are ultimately important for visual perception. Based on these results, we explore the potential roles of forelimb stripe coloration in painted turtles and discuss future studies that could determine the function(s) of coloration in this brightly colored reptile.

### Color and physiology

Color reflects physiological state across many taxa, particularly in males (e.g., birds, Hill and McGraw 2006; lizards, Plasman et al. 2015; frogs, Desprat et al. 2017). In turtles, multiple studies support a relationship between coloration and health. When red-eared slider turtles received an immune challenge, brightness of the yellow chin stripes decreased compared to control individuals (Ibáñez et al. 2014). An additional study detected a relationship between H: L ratio, an immune challenge, and coloration in red-eared slider turtles (Polo-Cavia et al. 2013). In painted turtles, despite measuring many aspects of physiology, we find no strong relationships between stress or immune measures and coloration of the forelimb stripes. The single relationship found between lymphocyte stimulation index in response to lipopolysaccharide (LPS) and chromatic contrast was driven by one outlier individual, suggesting this relationship may not be biologically meaningful. This lack of relationship between physiology measures and color is surprising given previous work in turtles and the large number of studies that have detected relationships between carotenoid-based coloration and aspects of health in vertebrates (Svensson and Wong 2011; Hill et al. 2023). However, there are recent examples of vertebrates where coloration is not associated with physiological health. In another study on the same painted turtle population studied here, no relationship was found between bactericidal competence of plasma and coloration (Stasiek and Reinke 2025). A meta-analysis of bird feather coloration found that color was not a significant predictor of immune function (Weaver et al. 2018b). Recent work has even challenged the assumption that carotenoids are costly or provide benefits to physiological function (Koch et al. 2018, 2019).

Ultimately, if coloration is a condition-dependent (honest) signal, it should reflect some aspect of quality in the wild. Carotenoid signaling may not need to be the result of a costly trade-off between physiological function and ornamentation, but may instead be an honest signal if carotenoid pigmentation is inherently tied to fundamental physiological processes (the shared pathway hypothesis; Hill et al. 2023). One such process could be mitochondrial function, particularly when converting dietary carotenoids to modified carotenoids (e.g., red ketocarotenoids) is required to produce specific pigments, as is the case for turtles (reviewed in Powers and Hill 2021; Hill et al. 2023). While other aspects of physiology (e.g., stress responses, immune function) are impacted by mitochondrial function (Hill 2014), the indirect links of carotenoid production to these aspects of physiology may not yield strong correlations between coloration and baseline circulating stress indicators or immune function. Directly measuring aspects of mitochondrial function (e.g., mitochondrial performance, metabolic rate) and manipulating mitochondrial function may better elucidate connections between health and carotenoids in painted turtles (Hill et al. 2019).

While the bright coloration of painted turtles could be an honest signal used in mate choice (e.g., Steffen et al. 2019) given the presence of male courtship displays, carotenoid coloration may be unrelated to sexual selection. Painted turtle populations in the northern and western extent of the range display the most dramatic coloration on the plastron in the species. These populations also exhibit extreme anoxia tolerance and cold tolerance (e.g., Storey et al. 1988; Fanter et al. 2020). Tolerance of these extreme physiological stressors requires reduced metabolic activity and tolerance of oxidative stress upon reoxygenation. Many studies have demonstrated that carotenoids are antioxidants (reviewed in Pérez-Rodríguez 2009), and antioxidants could be important in anoxia tolerance of turtles (Krivoruchko and Storey 2010). Carotenoids stored in the shell and skin could buffer hatchlings and adults from oxidative damage elicited during anoxia (Reinke et al. 2017). Thus, painted turtle coloration may serve a similar function to that of *Tigriopus* copepods. These copepods have red coloration produced by astaxanthin, a ketocarotenoid also found in painted turtle stripes, but this coloration is important for protection during oxidative challenges and is not driven by sexual selection (Weaver et al. 2018a; Powers et al. 2020). The methods used in this system, depriving individuals of carotenoids and then testing their responses to oxidative challenges, could be used to determine whether carotenoids help protect painted turtles against oxidative damage following anoxia. Further, the potential role of forelimb stripe coloration in mate choice should be investigated.

We detected no strong correlations among measured aspects of physiology, including baseline levels of stress indicators and innate and adaptive immune function. This outcome may be explained by the often condition-dependent nature of stress measures and their influence on immune function (reviewed in Johnstone et al. 2012; e.g., Gangloff et al. 2017). As we did not use turtles with visible signs of illness, we may not detect strong correlations among physiology measures that would be more apparent in the presence of large differences in health status (e.g., parasites, disease). Physiological responses also may change with age or sex in ways difficult to account for without larger sample sizes (e.g., Judson et al. 2020).

While we did not find any relationships among stress measures or immune function and coloration of the forelimb stripes of painted turtles, we cannot exclude the possibility that coloration does honestly signal quality. We lacked the ability to assess UV reflectance, which may be an important component of signaling health to conspecifics. Further, painted turtle visual modeling still relies on many assumptions, as most of our understanding of pond turtle vision is from the red-eared slider turtle, and even then, turtle vision violates many of the assumptions commonly made when modeling vision of other vertebrates (e.g., Rocha et al. 2008). Finally, measuring color of multiple parts of the body, including head and neck stripes and the plastron, may reveal condition-dependence in some aspects of coloration (e.g., Ibáñez et al. 2014; Stasiek and Reinke 2025).

### Color, size (age), and sex

Despite the lack of relationships among physiology and coloration, smaller, younger turtles had less bright, colorful forelimb stripes than larger turtles (Fig. 2). Size also predicts color in other turtle species, such as European pond turtles and Spanish terrapins, though the aspects of color influenced by size vary (Ibáñez et al. 2013, 2017). Given the potential shift in painted turtle male mating strategy from courtship displays to coercion with increasing male body size (Moldowan et al. 2020b), we expected that there may have been a similar shift in male allocation of carotenoids from signaling to self-maintenance with increasing size (and thus decreased chromaticity). Instead, we found that brightness and hue, and likely carotenoid allocation, increases with size for both sexes. This could be due to differences in diet between smaller and larger turtles; forage quality and availability have the potential to impact color, as both carotenoid supplementation and deprivation of painted turtle diets changes aspects of spot, stripe, and shell color (Steffen et al. 2019, 2021). Carotenoid availability in the environment is largely not considered to be limited in the wild (Koch and Hill 2018), but our experiment took place in semi-natural experimental ponds where resource limitation may be important. The experimental ponds were the same size, were given the same resources, and appeared to have similar environmental conditions including sunlight and plant growth (Judson, personal observation). Thus, any differences in diet may be due to competition for food resources, rather than forage quality. Interestingly, prior work in this population has shown that in wild-sampled turtles, immune function (natural antibody levels) increases with age (Judson et al. 2020), which may be the result of increased ability to acquire carotenoids with larger body size. Given this study did not find the same pattern of natural antibodies increasing with body size, future work should focus on understanding how potential trade-offs in carotenoid allocation may influence both immune function and coloration in a larger sample of turtles in a controlled experiment.

We also found a significant difference in chromatic contrast between males and females, with females showing greater differences in hue between stripe and background forelimb color than males (Table S4). This contrasts with prior work on stripe color, which found that males had redder forelimb stripes than females, though size was not accounted for (Rowe et al. 2014). As there appears to be sexual dichromatism in forelimb stripe coloration, we cannot rule out a role of forelimb stripe coloration in mate choice. It is interesting, however, that females are more colorful than males given males perform the courtship displays in painted turtles. Given the lack of UV measures in this study, which differ in painted turtle males and females (Steffen et al. 2021), we may not have fully detected sexual dichromatism that could be involved in mate choice.

## Conclusion

In sum, we found no strong relationships between any measured aspects of stress or immune function and coloration of forelimb stripes from the perspective of a turtle. However, we did find a relationship between body size and forelimb stripe coloration, which may reflect shifting allocation or acquisition of carotenoids with age. We also found relationships between body size, sex, and physiology measures. Finally, we have generated protocols for measuring multiple baseline stress indicators and innate and adaptive immune function in a turtle which can be used for future study of variation in wild populations. Future work should focus on expanding our understanding of how turtles view color and the function of carotenoid coloration in painted turtle fitness.

## Supplementary Information

Below is the link to the electronic supplementary material.

Supplementary Material 1 (DOCX 1.87 MB)

## Data Availability

The datasets generated and analyzed in this study, as well as the code to reproduce them, are available in Iowa State University’s DataShare Repository (10.25380/iastate.26014591).
