# Supplementary material for: Forelimb stripe coloration signals age, but not physiological health, in painted turtles
Source: Behav Ecol Sociobiol. 2025 Dec 29;80(1):5. doi: 10.1007/s00265-025-03679-0 (PMC12748116; doi:10.1007/s00265-025-03679-0)
Supplement: Supplementary file 1 — Supplementary Material 1 (DOCX 1.87 MB) [file 265_2025_3679_MOESM1_ESM.docx]

**Behavioral Ecology and Sociobiology**

**Supplementary Material for:**

Forelimb stripe coloration signals age, but not physiological health, in painted turtles

Jessica M. Judson^1,2,4,5^, Luke A. Hoekstra^1,3^, Kaitlyn G. Holden^1,4^, Anne M. Bronikowski^1,4^, Fredric J. Janzen^1,4,6^

^1^ Department of Ecology, Evolution, and Organismal Biology, Iowa State University, Ames, IA 50011, USA

^2^ Current address: Department of Biology, University of Wisconsin – La Crosse, La Crosse, WI 54601, USA

^3^ Current address: Department of Integrative Biology, Oklahoma State University, Stillwater, OK 74078, USA

^4^ Current address: W. K. Kellogg Biological Station, Departments of Fisheries and Wildlife & Integrative Biology, Michigan State University, Hickory Corners, MI 49060, USA

^5^ Correspondence e-mail: jjudson@uwlax.edu, judsonj2@msu.edu; ORCID: 0000-0002-0137-654X

^6^ Correspondence e-mail: [janzenf1@msu.edu](mailto:janzenf1@msu.edu); ORCID: 0000-0002-5919-196X

**Expanded Methods:**

Husbandry and Physiology Sampling

We captured painted turtles for this study using hoop nets in July 2014 from the Thomson Causeway Recreation Area (TCRA) in Thomson, IL in the United States. We transported these turtles ~300 km directly west to Iowa State University (ISU), where we housed turtles in 53-liter bins filled with dechlorinated water and set lighting to mimic the photoperiod of Ames, IA. We followed all applicable ISU IACUC guidelines for the care and use of animals in this study. Turtles brumated in cold rooms at 4°C in bins filled with water using a photoperiod mimicking that of winter photoperiod in Ames, IA during the winter of 2014. In April 2015, we seeded three semi-natural experimental ponds (19m x 15m x 1.5m; Mitchell et al. 2017) at the ISU Horticulture Research Station with 58 painted turtles (36 males, 22 females). Turtles lived in the ponds during the summer months of 2015, consuming aquatic plants, anurans, and invertebrates that colonized the ponds along with supplementary Mazuri® Aquatic Turtle Diet. We drained all ponds and returned turtles to ISU in October 2015 for overwintering, and turtles were released back into the ponds the following April.

In July 2016, at the conclusion of the nesting season, we drained all experimental ponds and removed the turtles. This period of time falls in the window of breeding activity, when females are not gravid and males are attempting mating, in at least some populations (Gist et al. 1990; Moldowan et al. 2020a). We measured plastron length and obtained a blood sample from the caudal vein of each turtle with a heparin-rinsed syringe to assess baseline measures of circulating corticosterone (CORT) and glucose. To ensure we were measuring baseline circulating CORT, we recorded handling time, which is the time from capture to completion of collection of blood, to compare with CORT measures, as CORT increases in the bloodstream within 10 minutes of handling in painted turtles (Polich 2016). We aliquoted whole blood (50µL) into tubes with 50µL of AIM V serum-free lymphocyte cell medium for lymphocyte proliferation assays (Palacios et al. 2013; Palacios and Bronikowski 2017) and made a blood smear stained with Wright Giemsa for differential cell counts. We centrifuged the remaining whole blood (from 30-150µL) and separated the plasma into two aliquots for CORT and immune assays before flash freezing in liquid nitrogen and storing at -80ºC.

Color Analysis

We took RAW-formatted photographs (tripod-mounted Canon EOS Digital Rebel XSi camera and EF-S18-55mm lens) of each turtle’s cranial region under controlled incandescent lighting and included a grey standard (18% reflectance; Insignia NS-DWB3M) in every photograph. These photographs were all taken on the same day one week after removal of turtles from the ponds. We took two photographs of each turtle at slightly different angles and checked photographs for overexposure before performing Quantitative Color Pattern Analysis (QCPA) in micaToolbox v. 2.3 (Troscianko and Stevens 2015; van den Berg et al. 2020) in ImageJ v. 1.54g (Schneider et al. 2012). micaToolbox linearizes photographs to account for slight variation in lighting conditions between photographs using a grey standard and calculates reflectances, which are then converted to cone-catch values using a model that accounts for the spectral sensitivities of the species of interest. These cone-catch values can then be used to analyze differences in luminance (the lightness and brightness), chromaticity, and achromatic and chromatic contrast elicited by and between specific regions of interest (ROIs) on the organism.

To analyze painted turtle color, we created a cone-catch model of painted turtle vision using the chart-based method of micaToolbox. We photographed a X-Rite ColorChecker Passport Photo 2 (X-Rite; Michigan, USA) with the aforementioned camera and lens under two conditions: outdoor sunlight, and incandescent lighting, under which the turtle photographs were taken. We then developed cone-catch models under both lighting conditions using a custom spectral sensitivity profile created from two turtle species. We obtained a long wavelength (LW) spectral sensitivity curve from a flicker photometry study in adult painted turtles (see Fig. 3 in Graf 1967) and medium wavelength (MW), short wavelength (SW), and double cone spectral sensitivity curves from intracellular retinal preparations of adult red-eared slider turtles (*Trachemys scripta elegans*; see Fig. 2 in Ohtsuka 1985), as there were no spectral sensitivity curves in those wavelength ranges for painted turtles. Though painted turtles also likely have cones sensitive to ultraviolet (UV) wavelengths given their presence in the closely related red-eared slider turtle (Zana et al. 2001), we did not have the capability to photograph UV reflectance measures in this study, and thus exclude any spectral sensitivities in the UV range. The spectral sensitivity research of Graf (1967) and Ohtsuka (1985) do not have data available online, so we used the figures and the online software WebPlotDigitizer v. 4.5 (Rohatgi 2020) to assign points along the curves at every value of wavelength from 400nm to 700nm. These values were then back transformed and re-scaled such that the area under the curve was equal to 1 for use in micaToolbox. The cone-catch models with our custom turtle spectral sensitivity file and the two chart photographs in either sunlight or incandescent light achieved recommended R^2^ values (all R^2^ > 0.98; <http://www.empiricalimaging.com/knowledge-base/chart-based-cone-catch-model/>). While the photographs were taken under non-natural lighting conditions, we wanted to test the applicability of the results to wild painted turtles in natural conditions. Thus, we measured the color patches of the X-Rite ColorChecker Passport Photo 2 using the model created with the sunlight chart photograph (the model representing natural conditions; hereafter the “daylight model”) in two scenarios. First, we measured color patches in the chart photograph taken in sunlight using the daylight model. Second, we measured those same color patches in a chart photograph taken in incandescent lighting conditions using the daylight model. Finally, we calculated root mean squared error (RMSE) between these two sets of color patch measurements for each channel of wavelengths in micaToolbox (SW, MW, LW, double cones) to assess the agreement of the values in these two illuminants (J. Troscianko, personal communication). RMSE ranged from 0.044 to 0.08, suggesting that there is only a small amount of disagreement between the two illuminant types for the colors measured. Thus, while we use the cone-catch model generated using a chart photograph taken under incandescent lighting conditions for QCPA, the results of this work can be applied to natural lighting conditions as well.

Both photographs of each turtle were converted to multispectral images (to standardize lighting conditions across photographs), which were then converted to cone-catch values with the incandescent light model and turtle spectral sensitivities described above. The double cone channel was treated as the luminance channel, as it is suggested that the function of double cones is more important for luminance than color vision in birds (e.g., Vorobyev et al. 1998) and lizards (Olsson et al. 2013), and non-avian and avian reptiles share many similarities in visual anatomy (reviewed in Osorio 2019). We used these cone-catch images to perform QCPA for analysis of differences in luminance and chromaticity (colorfulness) of the stripe among turtles, as well as differences in achromatic and chromatic contrast of the stripe when compared to the background forelimb color. While QCPA in micaToolbox provides many tools which use the Receptor Noise Limited Model (RNL, Vorobyev and Osorio 1998), current knowledge of turtle vision suggests that pond turtles may violate the assumptions of the RNL model, particularly with respect to color opponent processing (Rocha et al. 2008). Thus, to cluster the turtle’s forelimb into a stripe ROI and a “background” ROI, we performed clustering using the Naïve Bayes Clustering tool. To perform this analysis in micaToolbox v. 2.3, please reach out to J. Troscianko for a bug fix that caused this clustering to result in reports of standard deviations instead of mean cone catches. The stripe ROI in this study was the red, orange or yellow main stripe that runs down the front of the turtle’s forelimb from the claw to the elbow, while the background ROI was the region of the forelimb surrounding the stripe (Fig. 1). These stripes are visible to conspecifics during male courtship displays and aggression (Ernst and Lovich 2009; Moldowan et al. 2020b), and thus may serve as a signal of fitness to potential mates or competitors. To use the Naïve Bayes Clustering tool within QCPA, we selected as much of the right forelimb stripe as possible without overlapping the dark skin surrounding the stripe with the Selection Brush Tool in ImageJ and saved as the “stripe ROI” (Fig. S1a). We then selected a portion of the dark color of the forelimb surrounding the stripe and saved as the “background ROI” (Fig. S1b). Finally, we used the Polygon Selection Tool in ImageJ to select the forelimb region from claw to elbow surrounding the stripe and saved this as the ROI for performing clustering (Fig. S1c). Stripe color measures were similar for both forelimbs within an individual; thus, when the right forelimb stripe was not visible or was blurred, the left forelimb was measured instead. Once all ROIs were created in a photo, we calculated the linear normalized cone-catch values for the stripe and background ROIs in a table for use with the Naïve Bayes Clustering tool.

The QCPA framework requires many settings which we outline here for reproducibility. First, we chose a Gaussian acuity correction to measure the forelimb ROIs specifically, independent of the photograph background. We used Naïve Bayes clustering, a luminance Weber fraction of 0.1 (as recommended by <http://www.empiricalimaging.com/knowledge-base/running-the-qcpa-framework/> when behavioral validation is not available), and chose custom visual system Weber fractions. We calculated these fractions from abundance of each wavelength sensitive cone type in red eared slider turtles (LW=0.29%, MW=21%, SW=12%; Grötzner et al. 2020). Assuming a receptor noise of 0.05, Weber fractions were calculated as 0.05:0.06:0.08 for LW:MW:SW (<http://www.empiricalimaging.com/knowledge-base/cone-ratios/>). The acuity units were cycles per degree, and we used 6.1 as the acuity value, which was the average acuity in cycles per degree across tested red eared slider turtles (range of 4.4 to 9.9; Northmore and Granda 1991). We used a viewing distance of 500mm given the close interactions that occur during painted turtle displays, rescaled to 5 pixels per MRA (default setting), and chose the ROI described above that included the turtle’s forelimb from claw to elbow. This resulted in a table of average cone-catch values for each class of wavelength (LW, MW, SW, double cones) and *D_max_*, which is a measure of the maximum possible chromaticity that could be elicited by a color patch in a hypothetical opponent process (Endler and Mielke 2005; van den Berg et al. 2020), for both stripe and background regions of the forelimb ROI found after clustering. We used the average cone-catch values of the double cones to describe luminance of the forelimb stripe. *D_max_* was used to describe chromaticity given the complex color opponency of turtle vision (Rocha et al. 2008), which may violate assumptions used by the RNL to understand perceived color differences through opponent processes. In all cases of both the stripe and the background regions detected after clustering, the maximum chromaticity was between LW:SW, and thus *D_max_* was calculated as $\frac{LW-SW}{LW+SW}$ (van den Berg et al. 2020). Larger values of *D_max_* indicate greater maximum possible chromaticity (colorfulness) of a stripe.

Turtle vision may violate assumptions of the RNL model, given the large number of opponent responses found in red eared slider turtles (Rocha et al. 2008), but the visual system may also violate assumptions of *D_max_*. Specifically, more than two cones may be involved in a given opponent channel, and the high number of opponent channels found in red eared slider turtles suggests this may be a possibility (Rocha et al. 2008). Additionally, there are currently no methods to easily compare the chromaticity of two regions with *D_max_*, which means it can be difficult to interpret the ecological relevance of the signal to a receiver. Therefore, we also measured the achromatic (∆L) and chromatic (∆S) contrast between the stripe region and background region of the forelimb ROI using RNL methods in micaToolbox (Luminance and Colour JND Calculators; Troscianko and Stevens 2015). We first created a table in the format required using the average cone-catch values for each class of wavelength from the clustering and QCPA done above, using the double cones as the luminance channel. As before, we used a luminance Weber fraction of 0.1 and Weber fractions of 0.05 for LW, 0.06 for MW, and 0.08 for SW. These values can be interpreted similarly in that higher values indicate a greater contrast in brightness (∆L) or hue (∆S), and thus greater ability to distinguish the stripe from the background. Further, the results can also be interpreted in the form of “just noticeable differences”, such that a value greater than 3 suggests that the two regions could be distinguished in a natural setting by the viewer (Vorobyev and Osorio, 1998).

We assessed repeatability (intraclass correlation coefficient, Lessells and Boag 1987) of average cone-catch values, *D_max_*, ∆L, and ∆S using the two photos of each turtle in R v. 4.4.2 (R Core Team 2024). Stripe luminance, *D_max_*, ∆L, and ∆S were repeatable across photographs of the same turtle, with average intraclass correlations of 82%, 91%, 79%, and 91%, respectively. We averaged each of these measures across the two photographs of each turtle to produce final color variables used in statistical analyses. To assess whether color analysis of the forelimb stripe using the RNL model or the *D_max_* model led to different results, we assessed correlations among all average cone-catch values, *D_max_*, ∆L, and ∆S. Finally, we performed a principal components analysis (PCA) of stripe color variables, including all average cone-catch values for LW, MW, SW, and double cone (luminance) channels, to assess axes of color variation across turtles. Variables were centered and scaled before performing PCA with ‘prcomp’ in R. All data used for building turtle visual models, raw photographs, multispectral images, R scripts, and final color variables can be found in the repository ([https://figshare.com/s/0af5bbadd0cd59aa0d72](https://urldefense.com/v3/__https:/figshare.com/s/0af5bbadd0cd59aa0d72__;!!HXCxUKc!2cDLfxL_WwojlN9dT2l7VaoA-DV5oOZbjdE_wRcax7rE4PfQXu0FsSVu4Rr36J8GXgB-9SkU0py9lrL0ZzxpQCJYBKs7$)).

Physiological Measures

*Baseline Stress Indicators: Corticosterone, Glucose, and Heterophil:Lymphocyte Ratios*

The release of CORT induces increases in circulating glucose (Landys et al. 2006), increases in heterophils (neutrophils in mammals), and decreases in lymphocytes in the bloodstream to prepare the organism to appropriately respond to stressors (reviewed in Goessling et al. 2015). We quantified the baseline concentration of circulating plasma CORT (ng/mL) using a double-antibody radioimmunoassay (ImmuChem Double Antibody Corticosterone I-125 RIA kit, MP Biomedicals, Irvine, CA, USA). This assay follows previously described protocols validated in painted turtles (Refsnider et al. 2015; Polich 2016). We ran all samples (N=58) in duplicate, with a pooled sample included with each batch to assess inter-assay variability. CORT was not correlated with handling time (Pearson’s *r* = 0.18, *P* = 0.18). Turtles in this study did not appear to be more stressed than those living at TCRA, as baseline CORT values were similar to those reported across multiple studies at the TCRA (mean CORT this study=12.49 ng/mL; Refsnider et al. (2015) back-transformed least-square means=7 ng/mL; Polich (2016)=27.45 ng/mL).

Circulating glucose concentrations are produced by antagonism between glucocorticoids and insulin (Strack et al. 1995); appropriate concentrations of glucose are essential to both homeostatic functions and stress responses. We measured the baseline concentration of circulating glucose (Mg/dL) using 1.5μL blood plasma with a FreeStyle Lite® glucometer (Abbott Diabetes Care, Alameda, CA) and FreeStyle Lite® test strips (N=56; Gangloff et al. 2017).

Leukocyte profiles such as heterophil:lymphocyte (H:L) ratios measure physiological response to stressors (Davis et al. 2008; Polo-Cavia et al. 2013). Here, we analyzed H:L ratios via stained blood smears by identifying 100 leukocytes at 1000x magnification and counting the number of heterophils and lymphocytes within those leukocytes (N=55; Gangloff et al. 2017). Under chronic stress conditions, a large H:L ratio is produced by glucocorticoids mobilizing lymphocytes into tissues and out of the bloodstream, while heterophils are increased in the bloodstream (Davis et al. 2008).

*Innate Immune Function: Bactericidal Competence of Plasma, Natural Antibodies, and Lysis*

The bactericidal competence (BC) of plasma measures constitutive innate immune function. Turtles with increased innate immune function are characterized by a high bacterial killing capacity, or competence, while individuals with depressed immune function may exhibit lower bactericidal competence (Matson et al. 2006). We assessed BC of plasma according to Palacios et al. (2011) and Refsnider et al. (2015) with a few modifications noted here. We diluted *Escherichia coli* working solution 1:160 with sterile phosphate-buffered saline (PBS) to produce a working solution containing approximately 300 colony-forming bacteria per 10μL. We prepared samples (N=55) with 10μL plasma, 90μL warm PBS, and 10μL *E. coli* working stock. We performed 3 controls for each set of samples, consisting of 100μL warm PBS and 10μL *E. coli* working stock. We incubated samples and controls for 20 minutes at 28ºC before plating each sample and control in duplicate, with 50μL on each plate. Finally, we incubated plates for 24 hours at 28ºC. We calculated proportion of bacteria killed as the mean number of bacterial colonies on the sample plates compared to the mean of the control plates, and we converted this to a proportion by subtracting from 1 (Palacios et al. 2011). We detected a batch effect due to decreased survivorship of the *E. coli* working stock over time, and thus standardized BC within each batch (3 batches over 3 days).

Natural antibodies (NAbs) and complement-mediated lysis (CL) are two additional measures of constitutive innate immunity; high levels of NAbs and CL activity indicate a higher level of innate immune defense (Matson et al. 2005). We assessed these immune measures using a haemolysis-haemagglutination assay modified from Matson et al. (2005) for use in reptiles (Palacios et al. 2011), specifically painted turtles (Schwanz et al. 2011; Refsnider et al. 2015). We added 10µL plasma to the first column of a 96-well plate, and performed serial two-fold dilutions with 10µL of plasma and PBS beginning with the second column. Thus, the first column represents undiluted plasma, with each subsequent column diluted by a factor of two. We then added 10µL of a 2% sheep red blood cell (SRBC) suspension to each well. We gently mixed plates on a shaker for 2 minutes before incubating plates for 60 minutes at 28°C and then immediately scoring titers. We estimated titers as –log_2_ of the highest dilution factor of plasma that showed agglutination or lysis for NAbs and CL measures, respectively. As some wells showed partial agglutination or lysis, we scored intermediate titers as half scores. We ran all samples (N=53) in duplicate, with the exception of plasma-limited samples (N=3), and averaged scores across duplicates. We included a positive control (anti-SRBC antibodies diluted to 1:16, Fisher # ICN55800) and negative control (PBS) in each plate. Lysing ability of plasma samples using this method were low for our study, similar to Schwanz et al. (2011), and fewer individuals had enough plasma remaining for this assay (N=47). When possible, we averaged scores across duplicates. Samples that showed no lysis with undiluted plasma received a score of zero.

*Adaptive Immune Function: Lymphocyte proliferative ability*

Lymphocyte proliferation assays measure an organism’s adaptive immune function by assessing the activation and proliferation of B- and T-lymphocytes in response to a mitogen; increased proliferation indicates a stronger immune response (Palacios et al. 2013). We gauged lymphocyte proliferation ability with a whole-blood mitogenic stimulation assay (Palacios et al. 2013; Palacios and Bronikowski 2017) performed within 24 hours of blood collection. We assayed samples (N=55) in triplicate in a 96-well plate format. We used two T-cell mitogens, concanavalin A (ConA) and phytohemagglutinin (PHA), and one B-cell mitogen, lipopolysaccharide (LPS). Detailed methods for mitogens and concentrations can be found in Palacios et al. (2013). Briefly, we incubated mitogen- and control-treated triplicates for 96 hours total at 28°C in a 7% CO_2_ humidified atmosphere, and we pulsed plates with tritiated [^3^H] thymidine (0.5 µCi/well) for the final 24 hours of incubation. We harvested triplicates with glass-fiber filters using a cell harvester (Combi Cell Harvester; Skatron Instruments, Sterling, VA) and quantified thymidine incorporation in counts per minute (cpm) using a liquid scintillation counter (Palacios et al. 2013; Palacios and Bronikowski 2017). The proliferative ability of lymphocytes is expressed as a stimulation index (SI; Palacios et al. 2013), which is a ratio that compares mean cpm of mitogen-stimulated samples and non-stimulated controls. Samples with no difference in stimulation compared to controls received an SI of one. To control for differences in the initial number of lymphocytes in each sample, we estimated total leukocyte counts using the indirect Phloxin B method (Campbell and Ellis 2007) with 0.1% phloxin stain (Vetlab Supply, Palmetto Bay, FL) and hemocytometer (Palacios et al. 2013). We used slide preparations from the H:L assay to calculate the starting number of lymphocytes by multiplying the total leukocyte count by the proportion of lymphocytes for each individual. For the three individuals for which we could not detect enough cells on the slide preparations, we averaged the proportion of lymphocytes across all individuals and used that proportion to calculate starting number of lymphocytes. To correct the SI for starting number of lymphocytes, as greater numbers of lymphocytes should increase SI, we performed a linear regression of starting number of lymphocytes versus SI for each mitogen in R. We used the residuals from those models as values for SI of the three mitogens. Raw data from all physiology assays can be found in the repository ([https://figshare.com/s/0af5bbadd0cd59aa0d72](https://urldefense.com/v3/__https:/figshare.com/s/0af5bbadd0cd59aa0d72__;!!HXCxUKc!2cDLfxL_WwojlN9dT2l7VaoA-DV5oOZbjdE_wRcax7rE4PfQXu0FsSVu4Rr36J8GXgB-9SkU0py9lrL0ZzxpQCJYBKs7$).

Statistical Analyses

We used R v. 4.4.2 (R Core Team 2024) for all statistical analyses described here. There were no strong outliers in the physiology variables, with the exception of one individual for SI_ConA_ and two individuals for SI_PHA_ and SI_LPS_. The two outliers for SI_PHA_ and SI_LPS_ were turtles that exhibited high proliferation responses to both mitogens, and thus may be the result of biological differences. All models were run both removing or including the outliers, and in the one case where model results changed with their exclusion, we discuss both model outcomes. We first calculated correlations among all physiological variables to assess our prediction that baseline stress measures and immune function measures are associated with one another. We used Kendall’s rank correlation coefficient (Kendall 1938) given the presence of skewed distributions and putative outliers in physiology measures, used only complete observations, and calculated *P* values with Holm’s correction (Holm 1979) using the ‘corr.test’ function of the “psych” R package (Revelle 2024). We then performed a PCA to assess major axes of variance in physiology measures among turtles. We centered and scaled all variables except BC, which was already centered and scaled to account for batch effects.

To test the hypothesis that color varies with body size and sex in painted turtles, we first standardized plastron length by sex (zPL) using a sex-specific z-transformation, as female painted turtles attain larger body sizes than males (Hoekstra et al. 2018). We then performed general linear models of forelimb stripe luminance, chromaticity (*D_max_*), achromatic contrast (∆L) of the stripe compared to the background forelimb color, or chromatic contrast (∆S) with zPL, sex, and the interaction of zPL and sex as predictors using ‘lm’ in R. We included the interaction between sex and zPL because recent research suggests that mating strategy of male painted turtles may shift from courtship to coercion with increasing body size (Moldowan et al. 2020b). We assessed the relationship between physiology measures and color measures using general linear models with ‘lm’. We calculated F statistics and assessed statistical significance from model outputs using type III sums of squares analysis of variance in the “car” package (Fox and Weisberg 2019). We assessed model assumptions with “DHARMa” (Hartig 2022) and “car”.

To test the influence of separate immune and stress measures, we ran four sets of models including either the baseline stress measures or the immune measures with stripe luminance, stripe chromaticity, achromatic contrast, or chromatic contrast as the dependent variable. For all models, we included the interaction between zPL and sex and the fixed effects of sex, the pond in which each turtle was kept, and zPL. Thus, the models for stress response included pond, sex, zPL, the interaction of sex and zPL, CORT, glucose, and H:L ratio. The models for immune measures included pond, sex, zPL, the interaction of sex and zPL, standardized BC, NAbs, CL, SI_ConA_, SI_PHA_, and SI_LPS_. When statistically significant (i.e., P < 0.05), marginal means and pairwise contrasts among factors were estimated from models using “emmeans” (Lenth 2023). Any significant model results were plotted as partial regressions, such that predictors were plotted against residuals of a model excluding that predictor to account for other predictor effects in the model. Finally, we assessed the relationship between physiology measures, sex and zPL, which is a proxy for age in painted turtles, using general linear models (Hoekstra et al. 2018). We graphed all plots using “ggplot2” (Wickham 2016). All data used for statistical analyses and associated R scripts for analysis and figures can be found in the repository ([https://figshare.com/s/0af5bbadd0cd59aa0d72](https://urldefense.com/v3/__https:/figshare.com/s/0af5bbadd0cd59aa0d72__;!!HXCxUKc!2cDLfxL_WwojlN9dT2l7VaoA-DV5oOZbjdE_wRcax7rE4PfQXu0FsSVu4Rr36J8GXgB-9SkU0py9lrL0ZzxpQCJYBKs7$)).

**Supplemental Table 1** Correlations of 58 painted turtle forelimb stripe average cone-catch values for each class of wavelengths (long wavelengths = LW, medium wavelengths = MW, short wavelengths = SW, and double cone values = DBL), chromaticity (*D_max_*) of the stripe, and achromatic (∆L) and chromatic (∆S) contrast of the stripe compared to the background forelimb color. Pearson’s *r* reported below the diagonal, *P* values corrected using Holm’s correction (Holm 1979) reported above the diagonal. Correlations with *P* < 0.05 in bold.

|  | LW | MW | SW | DBL | *D_max_* | ∆L | ∆S |
| --- | --- | --- | --- | --- | --- | --- | --- |
| LW | − | **0.0000** | **0.0000** | **0.0000** | 0.2575 | **0.0002** | 0.2677 |
| MW | 0.7370 | − | **0.0000** | **0.0000** | **0.0024** | **0.0073** | 0.1192 |
| SW | 0.6493 | 0.9407 | − | **0.0000** | **0.0000** | 0.0776 | **0.0043** |
| DBL | 0.9867 | 0.8369 | 0.7540 | − | 0.6686 | **0.0002** | 0.6569 |
| *D_max_* | 0.2018 | -0.4284 | -0.5993 | 0.0574 | − | 0.5370 | **0.0000** |
| ∆L | 0.5403 | 0.4241 | 0.3313 | 0.5386 | 0.1789 | − | **0.0029** |
| ∆S | 0.2548 | -0.3051 | -0.4476 | 0.1306 | 0.8741 | 0.4616 | − |

**Supplemental Table 2** Correlations among physiology measures and size for painted turtles with no missing measurements (N = 45). Kendall’s rank correlation coefficients are below the diagonal, *P* values corrected using Holm’s correction (Holm 1979) are above the diagonal. No correlations were significant at *P* < 0.05.

|  | SI_ConA_ | SI_PHA_ | SI_LPS_ | H:L | NAbs | CL | CORT | Glucose | BC | zPL |
| --- | --- | --- | --- | --- | --- | --- | --- | --- | --- | --- |
| SI_ConA_^a^ | − | 1 | 1 | 1 | 1 | 1 | 1 | 1 | 1 | 1 |
| SI_PHA_ | -0.1756 | − | 0.7551 | 1 | 1 | 1 | 1 | 1 | 1 | 1 |
| SI_LPS_ | -0.1146 | 0.3537 | − | 1 | 1 | 1 | 1 | 1 | 1 | 1 |
| H:L^b^ | 0.0575 | 0.0746 | -0.2239 | − | 1 | 1 | 1 | 1 | 1 | 1 |
| NAbs^c^ | -0.2082 | -0.0685 | 0.0194 | 0.0934 | − | 1 | 1 | 1 | 0.1244 | 1 |
| CL^d^ | -0.0375 | 0.1094 | 0.0075 | 0.2525 | 0.1987 | − | 1 | 1 | 1 | 1 |
| CORT^e^ | 0.0354 | 0.0110 | -0.0207 | 0.1763 | 0.3210 | 0.1964 | − | 1 | 1 | 1 |
| Glucose | -0.0587 | -0.0954 | 0.0782 | -0.1631 | 0.0376 | 0.0946 | 0.0722 | − | 1 | 1 |
| BC^f^ | -0.2561 | -0.0415 | -0.1073 | 0.1774 | 0.4359 | 0.2742 | 0.2306 | 0.0318 | − | 1 |
| zPL^g^ | -0.1867 | -0.0256 | -0.1208 | 0.1126 | -0.1346 | 0.1575 | -0.1661 | -0.0673 | 0.1452 | − |

^a^SI = lymphocyte stimulation index for respective mitogen (ConA, PHA, LPS) standardized by starting number of lymphocytes, ^b^H:L = heterophil:lymphocyte ratio, ^c^NAbs = natural antibodies, ^d^CL = complement-mediated lysis, ^e^CORT = corticosterone, ^f^BC = bactericidal competence standardized by batch, ^g^zPL = plastron length standardized by sex

**Supplemental Table 3** Models of painted turtle color vs size and sex including the interaction term

| Dependent Variable | Source of Variation | df | F | *P*-Value |
| --- | --- | --- | --- | --- |
| Luminance | zPL^a^ | 1, 54 | 1.4515 | 0.2335 |
|  | Sex | 1, 54 | 0.4964 | 0.4841 |
|  | zPL * Sex | 1, 54 | 0.0012 | 0.9721 |
| Chromaticity (*D_max_*) | zPL | 1, 54 | 2.9998 | 0.0890 |
|  | Sex | 1, 54 | 2.3427 | 0.1317 |
|  | zPL * Sex | 1, 54 | 0.1569 | 0.6936 |
| Achromatic Contrast (∆L) | zPL | 1, 54 | 1.0933 | 0.3004 |
|  | Sex | 1, 54 | 2.5316 | 0.1174 |
|  | zPL * Sex | 1, 54 | 0.0344 | 0.8536 |
| Chromatic Contrast (∆S) | zPL | 1, 54 | 7.2690 | **0.0093** |
|  | Sex | 1, 54 | 5.2494 | **0.0259** |
|  | zPL * Sex | 1, 54 | 0.5828 | 0.4485 |

df is degrees of freedom. *P* < 0.05 in bold. ^a^ plastron length standardized by sex

**Supplemental Table 4** Models of painted turtle color vs size and sex excluding the interaction term

| Dependent Variable | Source of Variation | df | F | *P*-Value |
| --- | --- | --- | --- | --- |
| Luminance | zPL^a^ | 1, 55 | 4.1260 | **0.0471** |
|  | Sex | 1, 55 | 0.5056 | 0.4801 |
| Chromaticity (*D_max_*) | zPL | 1, 55 | 5.4523 | **0.0232** |
|  | Sex | 1, 55 | 2.3791 | 0.1287 |
| Achromatic Contrast (∆L) | zPL | 1, 55 | 3.8582 | 0.0546 |
|  | Sex | 1, 55 | 2.5769 | 0.1142 |
| Chromatic Contrast (∆S) | zPL | 1, 55 | 11.7663 | **0.0012** |
|  | Sex | 1, 55 | 5.2895 | **0.0253** |

df is degrees of freedom. *P* < 0.05 in bold. ^a^ plastron length standardized by sex

**Supplemental Table 5** Relationship between baseline stress measures and painted turtle forelimb stripe luminance or chromaticity

| Dependent Variable | Source of Variation | df | F | *P*-Value |
| --- | --- | --- | --- | --- |
| Luminance | Pond | 2, 44 | 0.2759 | 0.7602 |
|  | Sex | 1, 44 | 0.1468 | 0.7034 |
|  | zPL^a^ | 1, 44 | 3.3441 | 0.0742 |
|  | CORT^b^ | 1, 44 | 0.0155 | 0.9015 |
|  | Glucose | 1, 44 | 0.3625 | 0.5502 |
|  | H:L^c^ | 1, 44 | 0.8738 | 0.3550 |
|  | Sex * zPL | 1, 44 | 0.0868 | 0.7697 |
| Chromaticity (*D_max_*) | Pond | 2, 44 | 0.4148 | 0.6630 |
|  | Sex | 1, 44 | 2.9332 | 0.0938 |
|  | zPL | 1, 44 | 4.3718 | **0.0424** |
|  | CORT | 1, 44 | 0.1003 | 0.7530 |
|  | Glucose | 1, 44 | 0.4359 | 0.5125 |
|  | H:L | 1, 44 | 2.3320 | 0.1339 |
|  | Sex * zPL | 1, 44 | 0.0030 | 0.9563 |

df is degrees of freedom. P < 0.05 in bold. ^a^ plastron length standardized for each sex; ^b^ corticosterone concentration; ^c^ heterophil:lymphocyte ratio

**Supplemental Table 6** Relationship between immune function measures and painted turtle forelimb stripe luminance or chromaticity

| Dependent Variable | Source of Variation | df | F | *P*-Value |
| --- | --- | --- | --- | --- |
| Luminance | Pond | 2, 33 | 0.3266 | 0.7236 |
|  | Sex | 1, 33 | 4.2133 | **0.0481** |
|  | zPL^a^ | 1, 33 | 1.3291 | 0.2572 |
|  | NAbs^b^ | 1, 33 | 0.5397 | 0.4677 |
|  | CL^c^ | 1, 33 | 0.9000 | 0.3497 |
|  | BC^d^ | 1, 33 | 0.0700 | 0.7930 |
|  | ConA^e^ | 1, 33 | 0.3250 | 0.5725 |
|  | PHA^f^ | 1, 33 | 0.6344 | 0.4314 |
|  | LPS^g^ | 1, 33 | 0.0728 | 0.7890 |
|  | Sex * zPL | 1, 33 | 0.9023 | 0.3491 |
| Chromaticity (*D_max_*) | Pond | 2, 33 | 0.0241 | 0.9762 |
|  | Sex | 1, 33 | 0.5372 | 0.4688 |
|  | zPL | 1, 33 | 4.2280 | **0.0477** |
|  | NAbs | 1, 33 | 0.2979 | 0.5889 |
|  | CL | 1, 33 | 0.2731 | 0.6048 |
|  | BC | 1, 33 | 0.5653 | 0.4575 |
|  | ConA | 1, 33 | 2.0824 | 0.1584 |
|  | PHA | 1, 33 | 2.2680 | 0.1416 |
|  | LPS | 1, 33 | 2.4362 | 0.1281 |
|  | Sex * zPL | 1, 33 | 0.0358 | 0.8511 |

df is degrees of freedom. P < 0.05 in bold. ^a^ plastron length standardized by sex; ^b^ natural antibodies; ^c^ complement-mediated lysis; ^d^ bactericidal competence standardized by batch; ^e^ residuals of models of lymphocyte proliferative responses to concanavalin A, ^f^ phytohemagglutinin, and ^g^ lipopolysaccharide against starting number of lymphocytes

**Supplemental Table 7** Models of painted turtle physiology measures versus size and sex

| Dependent Variable | Source of Variation | df | F | *P*-Value | Direction |
| --- | --- | --- | --- | --- | --- |
| SI_ConA_^a^ | zPL^g^ | 1, 52 | 0.3608 | 0.5507 |  |
|  | Sex | 1, 52 | 0.1188 | 0.7317 |  |
| SI_PHA_ | zPL | 1, 52 | 0.0595 | 0.8082 |  |
|  | Sex | 1, 52 | 0.2518 | 0.6179 |  |
| SI_LPS_ | zPL | 1, 52 | 0.1995 | 0.6570 |  |
|  | Sex | 1, 52 | 0.0005 | 0.9821 |  |
| H:L^b^ | zPL | 1, 52 | 1.2520 | 0.2683 |  |
|  | Sex | 1, 52 | 3.8125 | 0.0563 | M>F |
| NAbs^c^ | zPL | 1, 50 | 4.2386 | **0.0447** | Decrease |
|  | Sex | 1, 50 | 2.7241 | 0.1051 | M>F |
| CL^d^ | zPL | 1, 44 | 1.0833 | 0.3037 |  |
|  | Sex | 1, 44 | 12.4085 | **0.0010** | M>F |
| CORT^e^ | zPL | 1, 55 | 6.7558 | **0.0120** | Decrease with size |
|  | Sex | 1, 55 | 2.4889 | 0.1204 |  |
| Glucose | zPL | 1, 53 | 0.2140 | 0.6456 |  |
|  | Sex | 1, 53 | 0.1026 | 0.7499 |  |
| BC^f^ | zPL | 1, 52 | 2.7672 | 0.1022 | Increase |
|  | Sex | 1, 52 | 0.1180 | 0.7326 |  |

^a^SI = lymphocyte stimulation index for respective mitogen (ConA, PHA, LPS) standardized by starting number of lymphocytes, ^b^H:L = heterophil:lymphocyte ratio, ^c^NAbs = natural antibodies, ^d^CL = complement-mediated lysis, ^e^CORT = corticosterone, ^f^BC = bactericidal competence standardized by batch, ^g^zPL = plastron length standardized by sex. df is degrees of freedom. *P* < 0.05 in bold

**Fig S1** Example of region of interest (ROI) selection for the Naïve Bayes Clustering tool of micaToolbox using the nonlinear color image viewing of a multispectral image. a) manual selection of the forelimb stripe, b) selection of background region, c) selection of ROI containing the stripe and background region surrounding the stripe, which was ultimately used to cluster the region into the stripe and background cone-catch values

**Fig S2** First two principal components of painted turtle forelimb stripe average cone-catch values for each class of wavelengths (long wavelengths = LW, medium wavelengths = MW, short wavelengths = SW, and double cone values = DBL), which account for 99% of the variance in color variables. Individuals colored by sex (F = female, M = male). Eigenvectors shown with arrows

**Fig S3** Scree plot of percentage of variance explained by each principal component of painted turtle physiology measures

**Fig S4** First two principal components of painted turtle physiology measures, which account for 44% of the variance. Individuals colored by sex (F = female, M = male). Eigenvectors shown with arrows. ConA, PHA, LPS = lymphocyte stimulation index for respective mitogen standardized by starting number of lymphocytes, H:L = heterophil:lymphocyte ratio, NAbs = natural antibodies, CL = complement-mediated lysis, CORT = corticosterone, BC = bactericidal competence standardized by batch

**Fig S5** Partial regression plots showing the impact of outlier inclusion on model results of lymphocyte stimulation index in response to lipopolysaccharide (LPS) or concanavalin A (ConA) and painted turtle forelimb chromatic contrast (∆S). Lymphocyte stimulation index for respective mitogen is standardized by starting number of lymphocytes. Lines of best fit between lymphocyte stimulation index in response to mitogen (LPS or ConA) and residuals of a model excluding that mitogen response are shown.

References:

Campbell T, Ellis CK (2007). Avian and exotic animal hematology and cytology. 3rd edn. Blackwell Publishing, Ames

Davis AK, Maney DL, Maerz JC (2008) The use of leukocyte profiles to measure stress in vertebrates: a review for ecologists. Functional Ecology 22:760-772 <https://doi.org/10.1111/j.1365-2435.2008.01467.x>

Endler JA, Mielke PWJ (2005) Comparing entire colour patterns as birds see them. Biological Journal of the Linnean Society 86:405-431

Ernst CH, Lovich JE (2009). Turtles of the United States and Canada. 2nd ed. Johns Hopkins University Press, Baltimore

Fox J, Weisberg S (2019). An R companion to applied regression. 3rd Sage Publications, Thousand Oaks, CA

Gangloff EJ, Sparkman AM, Holden KG, Corwin CJ, Topf M, Bronikowski AM (2017) Geographic variation and within-individual correlations of physiological stress markers in a widespread reptile, the common garter snake (*Thamnophis sirtalis*). Comparative Biochemistry and Physiology, Part A: Molecular & Integrative Physiology 205:68-76 <https://doi.org/10.1016/j.cbpa.2016.12.019>

Gist DH, Michaelson JA, Jones JM (1990) Autumn mating in the painted turtle, Chrysemys picta. Herpetologica:331-336

Goessling JM, Kennedy H, Mendonça MT, Wilson AE, Grindstaff J (2015) A meta-analysis of plasma corticosterone and heterophil : lymphocyte ratios - is there conservation of physiological stress responses over time? Functional Ecology 29:1189-1196 <https://doi.org/10.1111/1365-2435.12442>

Graf V (1967) A spectral sensitivity curve and wavelength discrimination for the turtle *Chrysemys picta picta*. Vision Research 7:915-928 <https://doi.org/10.1016/0042-6989(67)90010-7>

Grötzner SR, de Farias Rocha FA, Corredor VH, Liber AMP, Hamassaki DE, Bonci DMO, Ventura DF (2020) Distribution of rods and cones in the red-eared turtle retina (Trachemys scripta elegans). Journal of Comparative Neurology 528:1548-1560 <https://doi.org/https://doi.org/10.1002/cne.24830>

Hartig F (2022). DHARMa: Residual Diagnostics for Hierarchical (Multi-Level / Mixed) Regression Models.

Hoekstra LA, Weber RC, Bronikowski AM, Janzen FJ (2018) Sex-specific growth, shape, and their impacts on life history of a long-lived vertebrate. Evolutionary Ecology Research 19:639–657

Holm S (1979) A simple sequentially rejective multiple test procedure. Scandinavian Journal of Statistics 6:65-70

Kendall MG (1938) A new measure of rank correlation. Biometrika 30:81-93

Landys MM, Ramenofsky M, Wingfield JC (2006) Actions of glucocorticoids at a seasonal baseline as compared to stress-related levels in the regulation of periodic life processes. General and Comparative Endocrinology 148:132-149 <https://doi.org/10.1016/j.ygcen.2006.02.013>

Lenth RV (2023). emmeans: Estimated Marginal Means, aka Least-Squares Means.

Lessells CM, Boag PT (1987) Unrepeatable repeatabilities: a common mistake. Auk 104:116-121 <https://doi.org/10.2307/4087240>

Matson KD, Ricklefs RE, Klasing KC (2005) A hemolysis-hemagglutination assay for characterizing constitutive innate humoral immunity in wild and domestic birds. Developmental & Comparative Immunology 29:275-286 <https://doi.org/10.1016/j.dci.2004.07.006>

Matson KD, Tieleman BI, Klasing KC (2006) Capture stress and the bactericidal competence of blood and plasma in five species of tropical birds. Physiological and Biochemical Zoology 79:556-564 <https://doi.org/10.1086/501057>

Mitchell TS, Refsnider JM, Sethuraman A, Warner DA, Janzen FJ (2017) Experimental assessment of winter conditions on turtle nesting behaviour. Evolutionary Ecology Research 18:271-280

Moldowan P, Brooks R, Litzgus J (2020a) Demographics of injuries indicate sexual coercion in a population of painted turtles (Chrysemys picta). Canadian Journal of Zoology 98:269-278

Moldowan PD, Brooks RJ, Litzgus JD (2020b) Sex, shells, and weaponry: coercive reproductive tactics in the painted turtle, Chrysemys picta. Behavioral Ecology and Sociobiology 74:1-14

Northmore D, Granda A (1991) Refractive state, contrast sensitivity, and resolution in the freshwater turtle, Pseudemys scripta elegans, determined by tectal visual-evoked potentials. Visual Neuroscience 7:619-625

Ohtsuka T (1985) Relation of spectral types to oil droplets in cones of turtle retina. Science 229:874-877

Olsson M, Stuart-Fox D, Ballen C (2013) Genetics and evolution of colour patterns in reptiles. Seminars in Cell & Developmental Biology 24:529-541 <https://doi.org/10.1016/j.semcdb.2013.04.001>

Osorio D (2019) The evolutionary ecology of bird and reptile photoreceptor spectral sensitivities. Current Opinion in Behavioral Sciences 30:223-227

Palacios MG, Bronikowski AM (2017) Immune variation during pregnancy suggests immune component-specific costs of reproduction in a viviparous snake with disparate life-history strategies. Journal of Experimental Zoology, Part A 327:513-522 <https://doi.org/10.1002/jez.2137>

Palacios MG, Sparkman AM, Bronikowski AM (2011) Developmental plasticity of immune defence in two life-history ecotypes of the garter snake, *Thamnophis elegans* - a common-environment experiment. Journal of Animal Ecology 80:431-437 <https://doi.org/10.1111/j.1365-2656.2010.01785.x>

Palacios MG, Cunnick JE, Bronikowski AM (2013) Complex interplay of body condition, life history, and prevailing environment shapes immune defenses of garter snakes in the wild. Physiological and Biochemical Zoology 86:547-558 <https://doi.org/10.1086/672371>

Polich RL (2016) Stress hormone levels in a freshwater turtle from sites differing in human activity. Conservation Physiology 4:1-9 <https://doi.org/10.1093/conphys/cow016>

Polo-Cavia N, López P, Martín J (2013) Head coloration reflects health state in the red-eared slider *Trachemys scripta elegans*. Behavioral Ecology and Sociobiology 67:153-162 <https://doi.org/10.1007/s00265-012-1435-z>

R Core Team (2024). R: A language and environment for statistical computing. R Foundation for Statistical Computing.

Refsnider JM, Palacios MG, Reding DM, Bronikowski AM (2015) Effects of a novel climate on stress response and immune function in painted turtles (*Chrysemys picta*). Journal of Experimental Zoology, Part A 323:160-168 <https://doi.org/10.1002/jez.1902>

Revelle W (2024). psych: Procedures for Psychological, Psychometric, and Personality Research.

Rocha F, Saito C, Silveira L, De Souza J, Ventura D (2008) Twelve chromatically opponent ganglion cell types in turtle retina. Visual Neuroscience 25:307-315

Rohatgi A (2020). WebPlotDigitizer.

Schneider CA, Rasband WS, Eliceiri KW (2012) NIH Image to ImageJ: 25 years of image analysis. Nature Methods 9:671-675 <https://doi.org/10.1038/nmeth.2089>

Schwanz L, Warner DA, McGaugh S, Di Terlizzi R, Bronikowski A (2011) State-dependent physiological maintenance in a long-lived ectotherm, the painted turtle (*Chrysemys picta*). Journal of Experimental Biology 214:88-97 <https://doi.org/10.1242/jeb.046813>

Strack AM, Sebastian RJ, Schwartz MW, Dallman MF (1995) Glucocorticoids and insulin: reciprocal signals for energy balance. American Journal of Physiology: Regulatory, Integrative and Comparative Physiology 268:R142-R149 <https://doi.org/10.1152/ajpregu.1995.268.1.R142>

Troscianko J, Stevens M (2015) Image calibration and analysis toolbox - a free software suite for objectively measuring reflectance, colour and pattern. Methods in Ecology and Evolution 6:1320-1331 <https://doi.org/10.1111/2041-210X.12439>

van den Berg CP, Troscianko J, Endler JA, Marshall NJ, Cheney KL (2020) Quantitative Colour Pattern Analysis (QCPA): A comprehensive framework for the analysis of colour patterns in nature. Methods in Ecology and Evolution 11:316-332 <https://doi.org/https://doi.org/10.1111/2041-210X.13328>

Vorobyev M, Osorio D (1998) Receptor noise as a determinant of colour thresholds. Proceedings of the Royal Society of London. Series B: Biological Sciences 265:351-358

Vorobyev M, Osorio D, Bennett AT, Marshall NJ, Cuthill IC (1998) Tetrachromacy, oil droplets and bird plumage colours. Journal of Comparative Physiology A 183:621-633

Wickham H (2016). ggplot2: Elegant Graphics for Data Analysis. Springer, New York

Zana Y, Ventura DF, De Souza JM, DeVoe RD (2001) Tetrachromatic input to turtle horizontal cells. Visual Neuroscience 18:759-765 <https://doi.org/10.1017/S0952523801185093>
